# Anxiety towards COVID-19, Fear of Negative Appearance, Healthy Lifestyle, and Their Relationship with Well-Being during the Pandemic: A Cross-Cultural Study between Indonesia and Poland

**DOI:** 10.3390/ijerph19127525

**Published:** 2022-06-20

**Authors:** Shally Novita, Dhini Andriani, Erika, Mariusz Lipowski, Małgorzata Lipowska

**Affiliations:** 1 Centre for Psychological Innovation and Research, Faculty of Psychology, Universitas Padjadjaran, Jl. Raya Bandung Sumedang KM 21, Sumedang 45363, Indonesia; dhini.andriani@unpad.ac.id; 2 Faculty of Nursing, Universitas Riau, Jl. Pattimura No. 9, Pekanbaru 28133, Indonesia; erika@lecturer.unri.ac.id; 3 Department of Psychology, Gdansk University of Physical Education and Sport, 1 Kazimierza Gorskiego Street, 80-336 Gdańsk, Poland; mariusz.lipowski@awf.gda.pl; 4 Institute of Psychology, University of Gdańsk, 80-309 Gdańsk, Poland; malgorzata.lipowska@ug.edu.pl

**Keywords:** well-being, anxiety towards COVID-19, fear of negative appearance, eating disorder, compulsive exercise

## Abstract

The COVID-19 pandemic has led to massive changes in almost all aspects of human life, including emotional states such as anxiety and fear, perspectives about healthy lifestyles, and psychological outcomes. This study aimed to disentangle the mechanisms that underlie the relationships of anxiety towards COVID-19 and fear of negative appearance with well-being, we also investigated the effects of cultural variations on levels of anxiety, fear of negative appearance, healthy lifestyles, and well-being. A total of 881 Indonesians (*n* = 172) and Poles (*n* = 709) participated in this study. Participants completed self-report measures of psychological well-being, anxiety, fear of negative appearance, compulsive exercise, and eating disorders. Multigroup structural equation modelling (SEM) was used. The results showed no statistically meaningful relationship between anxiety towards COVID-19 and well-being. However, it was found that, in the Polish sample, compulsive exercise and eating disorders mediated the relationship between fear of negative appearance and well-being. Cultural differences were also found in the mean scores of all examined constructs, with eating disorders being an exception. Therefore, this study highlights cultural aspects that determine emotional states, healthy lifestyles, and well-being.

## 1. Introduction

The COVID-19 pandemic has dramatically altered human activities, perceptions, and ways of life [1]. Many countries introduced policies such as lockdowns and social distancing to reduce transmission and prevent the spread of this virus. These massive changes disrupted daily living, with adverse psychological effects [2], including increased depression and anxiety, in addition to decreased quality of life and overall well-being [3]. In the last two years of the pandemic, many studies have examined well-being, but relatively few have sought to explain the mechanism underlying the relationships between fear, anxiety, and well-being, particularly in a cross-cultural context.

The inevitable physical repercussions of viral infections, huge changes in lifestyle, and restrictive regulations are likely to result in despair, tension, and anxiety [4,5,6]. Individuals with strong concerns about the pandemic may follow health guidelines exactingly, including prolonged social isolation. This may potentially affect psychological outcomes, including well-being and life satisfaction [7,8,9]. The ideal condition is to balance the implementation of health protocols with avoiding negative psychological outcomes [9]. This can be a challenge for individuals with high anxiety towards COVID-19.

There is a high likelihood that the fear of negative evaluation of one’s physical appearance has increased during the pandemic, and the rise in dissatisfaction with one’s appearance [10] can be attributed to frequent comparison with others through social media. In this respect, pre-pandemic studies have confirmed that there exist meaningful connections between fear of negative appearance and levels of body image, eating attitude, and mood after controlling for social physique anxiety [11]. This phenomenon is more prevalent among women than men [12]. The effect tends to be robust in adolescence and emerging adulthood [13]. Interestingly, the effects seem comparable across countries [14,15,16], indicating that the link between fear of negative appearance and well-being is very likely related to gender, but is not based on culture-specific factors.

Furthermore, previous studies reported that individuals in Indonesia had a tendency to engage in extreme diets and compulsive exercise prior to the pandemic [17]. High anxiety and fear correlate with extreme attitudes towards eating and physical exercise in the context of the need to stay healthy and physically fit during the outbreak. Previous studies reported that anxiety towards COVID-19 and fear of negative appearance change perceptions about healthy behaviors such as eating and physical activities. There is evidence that anxiety increases the risk of eating disorders. Individuals with low anxiety tend to engage in more healthy physical exercise than those with higher anxiety [4]. Similar results have been found regarding individuals with high levels of fear practicing unhealthy diets and performing compulsive exercise due to negative thoughts about their appearance [18].

Alongside anxiety and fear, there is evidence that a compulsively healthy lifestyle also impairs well-being. For example, a study in Hong Kong found that individuals with possible eating disorders reported lower levels of psychological well-being [19]. Individuals who have healthy eating habits and engage in the appropriate amount and intensity of physical activities are happier and more satisfied with their lives than those with unhealthy lifestyles [20]. The study also reported that such people have a more favorable perception of their health [20]. In general, good health is closely linked to well-being [21], and individuals with healthy lifestyles are in good health.

The relationships between anxiety towards COVID-19, fear of negative appearance, and well-being may be mediated by healthy behaviors. Anxieties and fears are states that may directly affect both behaviors and perceptions of one’s life satisfaction. Anxiety that is implemented in the form of behavior may strengthen one’s perception about their life satisfaction. Therefore, in addition to the direct effects of anxieties and fears on well-being mentioned above, there is a possibility that these relationships are mediated by healthy behaviors. Specifically, anxieties and fears change perceptions of healthy lifestyles and the associated practices. Individuals with high anxiety towards COVID-19 perceive the situation as a great threat to health and express anxiety in the form of extreme diet and physical exercise, which can be categorized as disorder behaviors [22,23]. In contrast, healthy lifestyle practices have been reported to be important antecedents of well-being [24].

Anxiety towards COVID-19, fear of negative appearance, healthy behaviors (e.g., eating habits), and physical exercise have significant links with psychological well-being [8,9,11]. These links have been predominantly established in Western European and North American populations. Fewer data are available from Southeast Asia and Eastern Europe. In addition, cross-cultural studies investigating a variety of countries are even more limited. This study compared individuals from various cultures and disentangled cultural views that determine variations in interpreting well-being, anxiety, fear of negative appearance, and healthy lifestyles. Indonesia and Poland were selected due to their distinct levels of cultural dimensions, such as Individualism (IDV) and the Uncertainty Avoidance Index (UAI) [25].

In an individualistic society, the ties between individuals are loose, and people identify themselves as “I” [25]. In a collectivistic society, individuals are part of a strong group, and people identify themselves as “we,” reflecting inclusion in a specific group [25]. These different cultural ideas impact the value placed on others’ perceptions, in particular, on fear of negative appearance.

The impacts of cultural values on well-being, anxiety towards COVID-19, fear of negative appearance, eating disorders, and compulsive exercise can be partly explained by the framework of cultural dimensions. However, it is still unclear whether cultural values may also influence the mechanism proposed by this study. Direct links between anxiety, fear of negative appearance, and well-being are consistent across studies [8,9], but the attempts to compare these effects to date have been insufficient. By comparison, the indirect links between the examined variables are relatively complex assumptions that may (or may not) be explained by cultural variations.

This study aimed both to disentangle the mechanisms that underlie the relationship between fears and anxiety and well-being during the pandemic, and to examine whether cultural differences also have effects on this mechanism. Compared to other studies conducted prior to COVID-19, this study examined this mechanism during the pandemic. This specific context may have a significant impact on the relationship between the above-mentioned constructs. In addition, specifically for Indonesia, this study is of great benefit.

Accordingly, this study assumed that anxiety, fear, and healthy lifestyles may have changed during the pandemic and these factors may affect well-being [8,9]. Drawing upon Hofstede’s framework of cultural dimensions [25], cultural expressions are observed in the different levels of well-being, anxiety, and fear of negative appearance. However, it is unclear whether cultural norms and values also influence the link between anxiety, fear, and well-being. Therefore, we formed the following hypotheses:

**Hypothesis** **1** **(H1).** *Anxiety towards COVID-19 has had a significant effect on well-being during the pandemic, and this effect is mediated by compulsive exercise and eating disorders. These relationships are still significant after controlling for age and education.*

**Hypothesis** **2** **(H2).** *Fear of negative appearance significantly affected well-being during the pandemic, and this effect is mediated by compulsive exercise and eating disorders. These relationships are still significant after controlling for age and education.*

**Hypothesis** **3** **(H3).** *Cultural differences can be observed in well-being, anxiety, and fear of negative appearance. Individuals from different countries are expected to express their cultural values in distinct levels of the abovementioned constructs.*

However, since it is unclear whether the relationships proposed in Hypotheses 1 and 2 will be different for societies with different values, the analysis thereof will use an exploratory approach.

## 2. Instruments and Methods

### 2.1. Sample

Participants aged 18 or older from Indonesia and Poland were invited to complete the survey using a snowball sampling procedure. In Indonesia, the authors asked both psychology and nursing students to fill out the questionnaires and spread them to their relatives and friends. In Poland, the author made an announcement via university websites and invited both students and the public to participate in the study and to spread the invitation to their friends and relatives. Data were collected between June 2020 and January 2021. During this time, several restrictions were implemented in Indonesia, particularly distance learning in the educational setting, while restrictions in Poland varied (in terms of restrictions in the educational setting and business sectors), depending on the number of COVID-19 cases. The participants were sent a link to a series of online questionnaires. Before they filled out the questionnaires, they were informed about the details of the study and had to provide their consent.

In Indonesia, *n* = 172 respondents with a mean age of 23.77 and SD of 7.73 participated in the study; around 91% of this sample were women. The total sample in Poland yielded *n* = 709 respondents with a mean age of 32.39 and SD of 11.38; 71% of respondents in Poland were women and 28% were men, while 1% identified themselves neither as male nor female.

### 2.2. Instruments

Before field implementation, the Psychological Well-Being Scale (PWBS), Coronavirus Anxiety Scale (CAS), Fear of Negative Appearance Evaluation Scale, and Obligatory Exercise Questionnaire were not fully available in Indonesian and therefore needed to be translated before they were used. The adaptation process followed the *Guidelines for the Process of Cross-Cultural Adaptation of Self-Report Measures* [26]. First, the items of the measurements were translated from English to Indonesian by two translators. Second, two researchers discussed the two resultant translations. Third, the final version, based on this discussion, was translated back to English by the two translators. Finally, the researchers discussed and compared the Indonesian version and the English version to decide the final items for each measurement.

All instruments except the PWBS, EAT, and CAS were also translated into Polish before field implementation using the above-mentioned procedure.

#### 2.2.1. Outcome Variable: Well-Being during the Pandemic

The Psychological Well-Being Scale designed by Ryff [27] was used to measure psychological well-being during the pandemic. The PWBS has been translated into more than 30 different languages [28]; therefore, in the presented research the Polish-language version by Karaś and Cieciuch [29] was used. This scale consists of six dimensions: self-acceptance, environmental mastery, positive relations, purpose in life, personal growth, and autonomy [30]. Each scale has three items, and each item uses a six-point Likert scale. The scores range from 1 (*strongly disagree*) to 6 (*strongly agree*). Before data analysis, several negative items were recoded. Therefore, a higher PWBS score indicates a higher degree of well-being. The psychometric properties of the PWBS are moderate, with a Cronbach’s alpha of 0.71 for Indonesia and 0.77 for Poland.

#### 2.2.2. Predictors and Mediators

##### Anxiety towards COVID-19

Level of anxiety towards COVID-19 was measured with the Coronavirus Anxiety Scale [31]. In Poland, the Polish translation developed by Skalski et al. [32] was used. The CAS aims to identify potential dysfunctional anxiety related to the pandemic. The participants were administered five items that assessed distinct physiological symptoms of fear and anxiety uniquely related to COVID-19. Each item used a five-point Likert scale, ranging from 1 (*not at all*) to 5 (*nearly every day over the past two weeks*). Higher scores indicate higher levels of anxiety towards COVID-19.

The CAS has been found to have relatively high reliability scores in several countries (e.g., 0.91–0.93 in China and 0.80 in Poland) [31,33]. In this study, Cronbach’s alpha was 0.87 for Indonesia and 0.81 for Poland.

##### Fear of Negative Appearance

Fear of negative appearance (FNA) is a variable that may identify the vulnerability of individuals to eating- and body-image-related problems [10]. In this study, the FNA was measured with the Fear of Negative Appearance Evaluation Scale (FNAES) [34]. The FNAES covers six items related to individuals’ concerns about others’ negative evaluations of their appearance. Each item uses a five-point Likert scale that ranges from 1 (*not at all*) to 5 (*extremely*). The psychometric properties are very good, with a Cronbach’s alpha of 0.90 for Indonesia and 0.95 for Poland.

##### Eating Disorders

Eating behavior was assessed using the Eating Attitude Test (EAT-26) [35], previously translated into many languages, including Polish [36]. This test was constructed to identify eating disorders, including anorexia nervosa. It consists of three dimensions: dieting (13 items), bulimia and food preoccupation (6 items), and oral and control behavior (7 items). In total, the EAT has 26 items scored on a six-point Likert scale ranging from 1 (*always*) to 6 (*never*). This instrument has been implemented in various countries and shown to have relatively high reliability scores. The reliabilities of the EAT in this study are 0.85 for both Indonesia and Poland.

##### Compulsive Exercise

Compulsive exercise [37] was examined with the Obligatory Exercise Questionnaire (OEQ). A prior study identified three dimensions of the OEQ: the emotional element of exercise, exercise frequency and intensity, and exercise preoccupation [38]. However, a US study conducted two decades ago recommended deleting 50% of the items based on the results of a factor analysis [38]. In this study, the three dimensions of the US study were implemented. The rest of the items were also included in three additional dimensions, resulting in six dimensions. The OEQ consists of 20 items that use a four-point Likert scale with scores ranging between 1 (*never*) and 4 (*always*). It had relatively high reliability in this study, with Cronbach’s alpha yielding 0.85 for Indonesia and 0.86 for Poland.

##### Sociodemographic Characteristics as Covariates

The following demographic characteristics were used as control variables: age and educational level. Educational level had six categories ranging from 1 (elementary school) to 6 (doctoral degree). 

### 2.3. Analytical Approach

The dimensions of the PWBS, EAT, and OEQ were used as indicators of well-being, eating disorders, and compulsive exercise, respectively. An item parceling approach (e.g., [39]) was used to determine the indicators of anxiety towards COVID-19, fear of negative appearance, and the last three dimensions of compulsive exercise. As a result, well-being and compulsive exercise each had six indicators, whereas fear of COVID-19, fear of negative appearance, and eating disorders each had three indicators.

Before the main analysis, the measurement invariance was checked between Indonesia and Poland [40]. This procedure was conducted in RStudio version 1.4.1106 [41] using the lavaan package version 0.6–8 [42]. The data were non-invariant; therefore, further analysis was performed. First, the modification indices were checked and then implemented in the model by deleting one indicator, such as freeing the constraints of compulsive exercise and anxiety, and specifying latent variables. The final model yielded a weak metric invariant between the Indonesian and Polish data.

A multigroup structural equation model (SEM) was utilized to examine the hypotheses. This analysis was performed in RStudio using the lavaan package version 0.6–8 [42]. Model fit was assessed with the root mean square error of approximation (RMSEA), comparative fit index (CFI), and Tucker–Lewis index (TLI) [43].

There were several steps in the testing of Hypotheses 1 and 2. First, well-being was regressed on all predictors: anxiety towards COVID-19, fear of negative appearance, eating disorders, compulsive exercise, age, and education. Second, the covariates, including the respondents’ age and education level, were specified in the model. Third, a bootstrap analysis with a random sample of 1000 was performed to rigorously test the indirect effects. In addition, to test Hypothesis 3, a series of model comparisons was performed by constraining specific parameters while freeing the rest.

## 3. Results

### 3.1. Descriptive Analysis

A descriptive analysis is presented in Table 1, and the large effect sizes between Indonesia and Poland are age, two indicators of well-being, and one indicator of compulsive exercise. Positive relation and purpose are two well-being indicators with relatively high effect size. This suggests that individuals from Poland have higher mean scores than those from Indonesia. Indicator 4 of compulsive exercise consists of items related to euphoria, art of exercises, and documentation of exercises.

### 3.2. Analysis of Measurement Model

In this study, a value of 0.50 was implemented as a cut-off score for accepted factor loadings [45]. Therefore, all factor loadings lower than 0.50 were excluded from further analysis. Three well-being indicators, one indicator of eating disorders, and two indicators of compulsive exercise were excluded from further analysis due to low factor loadings (Table 2). In addition, one indicator of compulsive exercise was dropped to ensure the metric invariant (see Section 2.3).

### 3.3. Analysis of Structural Model

Overall, the model satisfied the proposed criteria [46] with χ^2^ = 480.80, df = 186, CFI = 0.96, TLI = 0.95, RMSEA = 0.06, and SRMR = 0.06. The proportion between χ^2^ and df is located in an acceptable range between 2.00 and 5.00 [47,48]. The *R* square for well-being, the outcome variable, was 50% and 30% for Indonesia and Poland, respectively, indicating that the model explains a relatively high proportion of variance.

The analysis of the structural model, including bootstrapping analysis for indirect effects, could not confirm the relationship between anxiety towards COVID-19 and well-being. This result was similar both for Indonesian and Polish data, indicating that neither the effects of anxiety towards COVID-19 nor cultural differences between the two countries exist. Thus, our first hypothesis regarding the direct and indirect effects of anxiety towards COVID-19 and well-being cannot be confirmed.

**Figure 1:**
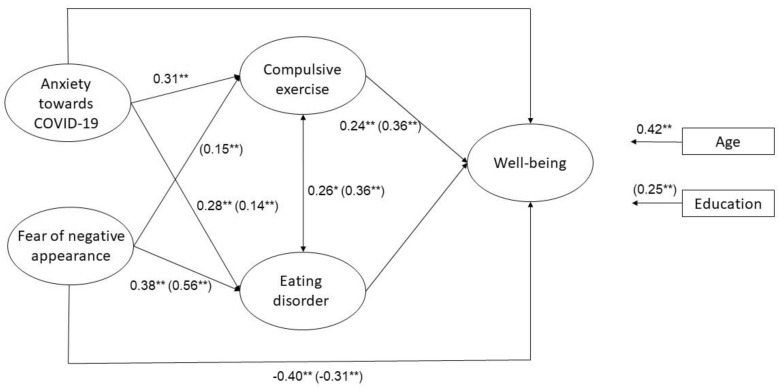
The structural equation model (SEM). Values represent the significant standardized regression coefficients. The values for Poland are in parentheses. ** *p* < 0.01; for more details on *p* values, see Appendix A.

The analysis of the indirect effect of fear of negative appearance on well-being yielded significant findings and this relationship was found to be mediated by compulsive exercise. However, this finding was only true for the Polish data. Although all direct relationships in the Indonesian data (see Figure 1) were found to be significant, the indirect effects found could not be distinguished from those obtained by chance. Therefore, Hypothesis 2 was partly confirmed. To provide the readers more information about the mediation hypotheses (Hypotheses 1 and 2), a summary of the indirect and total effects is presented in Table 3. A visualization of the model, including significant standardized coefficients, is presented in Figure 1. In addition, a report of all unstandardized coefficients, standard errors, and standardized coefficients of the model is available in Appendix A.

A series of model comparisons was performed to test Hypothesis 3. The latent mean comparison was conducted by comparing the model with free parameters and the model with some restrictions using an ANOVA approach. The latent mean comparisons yielded significant findings for well-being (*B* = −0.91, *SE* = 0.11, *p* = 0.000), anxiety towards COVID-19 (*B* = −0.37, *SE* = 0.10, *p* = 0.00), fear of negative appearance (*B* = 0.92, *SE* = 0.17, *p* = 0.00), and compulsive exercise (*B* = −2.11, *SE* = 0.19, *p* = 0.00). The analysis of eating disorders yielded an insignificant finding. These results indicate that individuals in Indonesia have lower well-being, anxiety, and compulsory exercise than those in Poland. They also confirm that fear of negative appearance is higher in Indonesia than in Poland.

Furthermore, results showed significant differences between Indonesia and Poland for the following paths: (1) anxiety towards COVID-19 and compulsive exercise; (2) fear of negative appearance and compulsive exercise; (3) fear of negative appearance and eating disorders; and (4) age and well-being. These results suggest that the effects of fear of negative appearance, anxiety, and healthy lifestyle on well-being are similar in Indonesia and Poland. However, the effects of fear of negative appearance and anxiety towards COVID-19 on compulsive exercise differ between the two countries. The model also detected differences between the two countries in the influence of fear of negative appearance on eating disorders, and the effect of age on well-being. Appendix A contains more information about the comparison of Indonesian and Polish models.

## 4. Discussion

This study examined a new paradigm about healthy lifestyles during COVID-19 and whether cultural differences regulate these emerging behavior patterns. It implemented relatively advanced and robust statistical analyses (e.g., [43]).

Analysis of the first hypothesis concerning the relationship between anxiety and well-being mediated by compulsive exercise and eating disorders yielded insignificant findings for both Indonesia and Poland, suggesting that the relationships between anxiety and well-being, including the indirect effects through compulsive exercise and eating disorders, are negligible. The pattern seems to be similar across countries that hold different cultural values.

Hypothesis 2 suggested that well-being during the pandemic was influenced by cultural norms (Table 3). However, both countries’ direct effects for fear of negative appearance and compulsive exercise on well-being were robust. These different results were obtained due to the insignificant link between fear of negative appearance and compulsive exercise in the Indonesian group (Figure 1). Polish participants expressed their fear of negative appearance through compulsive exercise to a greater extent than individuals from Indonesia. One explanation of this phenomenon is the high individualism (IDV) in Poland (IDV = 60, see [25]). In an individualistic culture, uniqueness is accepted and self-expression is valued [25]. Therefore, people from individualistic cultures can compensate for their fear of negative appearance by dieting and exercising. The low IDV in Indonesia (IDV = 14) may be related to low self-confidence in expressing negative perceptions of one’s own appearance. In a country with low IDV, collective perceptions are more important than an individual’s opinion [25]. Someone with high fear of a negative evaluation from others may not have the confidence to undertake physical exercise, particularly in public, due to possible negative feedback from one’s environment (see [49] for a discussion of the relationship between body image and social physique anxiety in a collectivistic country). A similar explanation may also explain the higher correlational magnitude between fear of negative appearance and eating disorders in Poland than in Indonesia.

Regarding Hypothesis 3, cultural differences were also found in the following four constructs: (1) anxiety towards COVID-19, (2) fear of negative appearance, (3) compulsive exercise and its relationship with anxiety, and (4) well-being. These findings are discussed below.

First, the comparative analysis found greater fear of negative appearance in Indonesia than in Poland. Others’ perceptions are more meaningful for Indonesians than for Polish people [25]. Therefore, the fear of negative appearance was higher. These results are in harmony with previous comparative studies between individualistic and collectivistic countries [50].

Second, individuals living in Indonesia had lower anxiety towards COVID-19 than did people from Poland. This reflects an individual’s anxiety towards external threats, mainly explained in the discourses about anxiety disorders. In this phenomenon, the differences between the Uncertainty Avoidance Index (UAI) of Poland (UAI = 93) and Indonesia (UAI = 48) affect the levels of anxiety of members of these societies [25]. The COVID-19 pandemic created uncertainty, which increased anxiety to a greater extent in countries with higher UAI [25].

Third, the Indonesians performed less compulsive exercise than did the Poles. Previous studies indicated that around 25% of an Indonesian sample [51], compared to 42% of a Polish sample [52], performed extensive physical exercise, including daily exercise. Possible explanations of these findings may include educational level and practical reasons for physical exercise, such as time constraints, motivation, and internalized beliefs about showing one’s body while exercising [46]. The stronger connection between anxiety towards COVID-19 and compulsive exercise in Indonesia may reflect an adaption to the pandemic. However, individuals from Indonesia reported less anxiety than did those from Poland, and their physical activity was higher during the pandemic [25]. Studies have shown that Polish people undertook less physical exercise during the pandemic than they did prior to the pandemic [53,54].

Fourth, Poles reported higher well-being levels than did Indonesians. A previous study found a higher level of well-being in individualistic countries than in collectivistic ones due to higher national income and better human rights practices.

Despite its important contribution to understanding well-being, this study has the following limitations. First, the sample characteristics of Indonesia and Poland were not similar. The size of the Indonesian sample was significantly smaller than the Polish one. Although multi-group SEM is robust towards this issue, a comparable sample size between the groups is preferable. It is worth noting that the Indonesian sample size is relatively small and the sampling was not randomized; therefore, inferences should be made cautiously. There was also a relatively high proportion of women in the Indonesian and Polish data. This limitation is very likely due to the starting point of the snowball sampling—psychology students and nursing faculties This limitation prevents a gender-specific analysis being performed on fear of negative appearance, eating disorders, and compulsive exercise. However, the literature suggests that there is a gender effect in these constructs [54,55]. Furthermore, any inferences about gender differences cannot be established and this was not the aim of this study. In addition, the Indonesian sample was younger, more homogenous, and less educated than the Polish one. These variables were controlled in order to better compare the samples. Second, the IDV and UAI indexes are based on dimensions proposed by Hofstede [25]. Although this cultural framework is very well known, the sample characteristics in Hofstede’s study may not be similar to those in this study.

## 5. Conclusions

This study examined both the mechanism that underlies the connection between general anxiety, fear, and well-being, and the manner in which cultural variations affect this mechanism. The results showed that compulsive exercise mediates the relationship between fear of negative appearance and well-being in Poland, but not in Indonesia. In addition, cultural variations were also observed in anxiety towards COVID-19, fear of negative appearance, and compulsive exercise, and in their relationship with well-being. These findings suggest that cultural norms and beliefs are expressed in various forms of both healthy behaviors and psychological outcomes.

## Figures and Tables

**Table 1:** Descriptive analysis of all variables.

Variable	Mean	SD	Min.	Max.	Effect Size (*d*)
Age	23.77 (32.39)	7.73 (11.38)	18 (18)	64 (74)	0.89
Education	3.44 (4.07)	0.80 (1.03)	2 (1)	6 (6)	0.68
Well-being ^1^					
Acceptance	12.60 (13.63)	2.17 (2.13)	7.00 (4.00)	18.00 (18.00)	0.48
Environmental mastery	14.13 (14.10)	2.01 (1.92)	9.00 (7.00)	18.00 (18.00)	0.01
Positive relations	11.31 (14.03)	2.59 (2.24)	4.00 (3.00)	17.00 (18.00)	1.12
Purpose in life	11.10 (13.32)	2.26 (2.18)	6.00 (4.00)	17.00 (18.00)	1.00
Personal growth	8.34 (8.93)	1.59 (1.61)	5.00 (6.00)	18.00 (18.00)	0.37
Autonomy	14.53 (14.46)	2.08 (2.07)	7.00 (5.00)	18.00 (18.00)	0.03
Anxiety towards COVID-19 ^1^					
Indicator 1	2.94 (3.28)	1.61 (1.76)	2.00 (2.00)	10.00 (10.00)	0.20
Indicator 2	2.55 (3.04)	1.23 (1.51)	2.00 (2.00)	10.00 (10.00)	0.20
Indicator 3	1.21 (1.24)	0.56 (0.63)	1.00 (1.00)	5.00 (5.00)	0.05
Fear of negative appearance ^1^					
Indicator 1	6.64 (5.40)	2.40 (2.13)	2.00 (2.00)	10.00 (10.00)	−0.55
Indicator 2	6.01 (5.12)	2.54 (2.24)	2.00 (2.00)	10.00 (10.00)	−0.37
Indicator 3	5.94 (4.96)	2.35 (2.34)	2.00 (2.00)	10.00 (10.00)	−0.42
Eating disorders ^1^					
Dieting	57.63 (56.29)	12.18 (9.98)	20.00 (23.00)	77.00 (78.00)	−0.12
Bulimia and food preoccupation	29.54 (31.01)	4.54 (3.90)	12.00 (9.00)	36.00 (36.00)	0.35
Oral and control behavior	30.97 (33.43)	5.31 (5.04)	15.00 (15.00)	42.00 (42.00)	0.48
Compulsive exercise ^1^					
Emotional element of exercise	6.47 (7.74)	2.15 (2.70)	4.00 (4.00)	14.00 (16.00)	0.52
Exercise frequency and intensity	7.70 (9.46)	2.32 (3.04)	4.00 (4.00)	15.00 (16.00)	0.65
Exercise preoccupation	3.38 (3.73)	1.31 (1.43)	2.00 (2.00)	8.00 (8.00)	0.26
Indicator 4	5.19 (7.13)	1.57 (2.36)	3.00 (3.00)	11.00 (11.00)	0.97
Indicator 5	9.01 (10.85)	2.42 (2.51)	5.00 (5.00)	17.00 (20.00)	0.75
Indicator 6	4.54 (4.58)	1.35 (1.30)	2.00 (2.00)	8.00 (8.00)	0.03

Note. *n*_Indonesia_ = 172, *n*_Poland_ = 709. Values for Poland are in parentheses. ^1^ Latent variables with indicators listed below. Numbering order of indicators (e.g., Indicator 1, 2, and 3) was created based on the item parceling procedure (see [39]). Cohen’s d criteria: small = 0.20; medium = 0.50, and large = 0.80 [44].

**Table 2:** Factor loadings of all indicators.

Variable	Factor Loadings
Well-being ^1^	
Acceptance	0.67 (0.74)
*Environmental mastery*	0.21 (0.64)
*Positive relations*	0.56 (0.47)
Purpose in life	0.72 (0.77)
Personal growth	0.63 (0.70)
*Autonomy*	0.42 (0.61)
Anxiety towards COVID-19 ^1^	
Indicator 1	0.71 (0.74)
Indicator 2	0.91 (0.96)
Indicator 3	0.86 (0.72)
Fear of negative appearance ^1^	
Indicator 1	0.83 (0.89)
Indicator 2	0.91 (0.95)
Indicator 3	0.89 (0.94)
Eating disorders ^1^	
Dieting	0.55 (0.76)
Bulimia and food preoccupation	0.80 (0.78)
*Oral and control behavior*	0.27 (0.48)
Compulsive exercise ^1^	
*Emotional element of exercise*	0.75 (0.69)
Exercise frequency and intensity	0.76 (0.87)
*Exercise preoccupation*	0.36 (0.65)
Indicator 4	0.75 (0.82)
Indicator 5	0.79 (0.80)
*Indicator 6*	0.29 (−0.16)

Note. *n*_Indonesia_ = 172, *n*_Poland_ = 709. Values for Poland are in parentheses. ^1^ Latent variables with indicators listed below. Indicators in italics were dropped. The first indicator of compulsive exercise, “Emotional element of exercise”, was dropped to ensure the invariant for both Indonesia and Poland (see Section 2.3). The rest of the indicators were dropped due to very low factor loadings.

**Table 3:** Regression coefficients.

Path	Indonesia		Poland	
	Indirect Effect	Total Effect	Indirect Effect	Total Effect
AC → CE → WB	0.06	0.02	0.01	−0.03
AC → ED → WB	−0.04	−0.08	−0.01	−0.05
FNA → CE → WB	−2.96	−3.15	0.03 **	−0.15 **
FNA → ED → WB	−0.03	−0.22 **	−0.03	−0.22 **

Note. *n*_Indonesia_ = 172, *n*_Poland_ = 709. AC = anxiety towards COVID-19, CE = compulsive exercise, ED = eating disorders, WB = well-being, FNA = fear of negative appearance. ** *p* < 0.00.

## Data Availability

Any inquiries and requests in this regard can be directed to the corresponding authors.

## Appendix A

**Table A1:** Structural Equation Model Analysis.

Path	Indonesia			Poland		
	B (SE)	*p*-Value	β	B (SE)	*p*-Value	β
AC → CE	0.48 (0.14)	0.00	0.31	0.06 (0.09)	0.47	0.03
AC → ED	1.79 (0.67)	0.01	0.28	0.82 (0.24)	0.00	0.14
AC → WB	−0.04 (0.07)	0.59	−0.05	−0.04 (0.04)	0.23	−0.05
FNA → CE	−0.14 (0.08)	0.08	−0.15	0.21 (0.18)	0.00	0.15
FNA → ED	1.42 (0.41)	0.00	0.38	2.42 (0.18)	0.00	0.58
FNA → WB	−0.19 (0.05)	0.00	−0.40	−0.19 (0.03)	0.00	−0.31
CE → WB	0.13 (0.05)	0.02	0.24	0.15 (0.02)	0.00	0.36
ED → WB	−0.02 (0.02)	0.15	−0.18	−0.01 (0.01)	0.17	−0.09
CE ↔ ED	2.80 (1.32)	0.03	0.26	5.89 (0.87)	0.00	0.36
Age → WB	0.05 (0.02)	0.00	0.42	0.00 (0.00)	0.23	0.05
Education → WB	0.02 (0.15)	0.89	0.02	0.27 (0.05)	0.00	0.25

Note. *n*_Indonesia_ = 172, *n*_Poland_ = 709. AC = anxiety towards COVID-19, CE = compulsive exercise, ED = eating disorders, WB = well-being, FNA = fear of negative appearance.
